# Anxiety and stress among Vietnamese health workers during the emergence of the SARS-CoV-2 Omicron variant: a cross-sectional study

**DOI:** 10.25122/jml-2024-0275

**Published:** 2024-10

**Authors:** Nguyen Van Hoat, Hoang Quynh Lien, Tran Quang Duc, Nguyen Bich Luu, Hoang Van Minh

**Affiliations:** 1 Quality Management Department, Hanoi Medical University Hospital, Ton That Tung, Dong Da, Hanoi, Vietnam; 2 Faculty of Technology, Dong Nai Technology University, Bien Hoa City, Vietnam; 3 Nursing Department, Hanoi University of Business and Technology, Vinh Tuy, Hai Ba Trung, Hanoi, Vietnam; 4 Center for Population Health Sciences, Hanoi University of Public Health, Duc Thang, Bac Tu Liem, Hanoi, Vietnam

**Keywords:** Anxiety, stress, health workers, COVID-19 pandemics, Vietnam

## Abstract

The prevalence and contributing factors of mental health issues among health workers in Vietnam during the early 2022 Omicron wave remain underexplored. This study investigated the prevalence and factors associated with anxiety and stress among health workers in a Vietnamese hospital during this period. A cross-sectional study, conducted from February 28 to April 14, 2022, at Hanoi Medical University Hospital, assessed anxiety and stress among 754 frontline health workers using the DASS-21 questionnaire via a Google survey. Logistic regression models were used to identify factors linked to anxiety and stress. Among healthcare workers, 22% experienced stress, and 33% anxiety. Multivariable logistic regression analysis revealed that direct contact with COVID-19 patients significantly increased the likelihood of experiencing stress (OR = 2.12, *P* < 0.01). Additionally, discrimination from relatives and friends was associated with higher odds of having anxiety (OR = 2.45, *P* < 0.001). Furthermore, a heavy workload significantly increased the odds of having anxiety (OR = 1.95, *P* < 0.001). Lastly, a lack of support from colleagues was linked to higher odds of experiencing stress (OR = 2.77, *P* < 0.05). The prevalence of mental health issues among Vietnamese health workers remained significant during the initial Omicron wave. Identified factors associated with these problems hold important policy implications for enhancing pandemic preparedness in Vietnam and worldwide.

## INTRODUCTION

At the onset of 2020, the emergence of the novel coronavirus disease (COVID-19) caused global upheaval, resulting in severe health and economic repercussions. Societies grappled with a rapid increase in cases and mortality rates, overwhelming healthcare infrastructures, significant economic pressures, and a surge in unemployment rates [1]. Additionally, from the beginning of the pandemic, concerns arose about the potential negative impacts on mental health stemming from COVID-19 [2]. The pandemic has markedly increased the need for medical care, making health workers particularly vulnerable to high levels of stress. Factors contributing to this stress include the overwhelming influx of confirmed or suspected patients, the constant risk of infection, excessive workloads, physical fatigue, and shortages of essential medications. These cumulative stressors can substantially precipitate detrimental mental health outcomes, both immediately and in the long term [3]. Healthcare practitioners, who constituted the frontline of the pandemic response, were already experiencing adverse mental health effects. Therefore, it was imperative to quickly assess the pivotal role of early detection of mental health issues and their subsequent effects while concurrently implementing measures to safeguard the psychological well-being of this demographic against the adverse impacts of the pandemic [4].

Amidst the COVID-19 pandemic, as evidenced by two distinct meta-analyses, the worldwide prevalence rates of anxiety spanned from 31.9% to 33.7% [5], respectively. These rates markedly exceeded the figures observed during the pre-COVID-19 era (2.5% to 7% for anxiety in 2017) [6]. Medical personnel, as the primary frontline against the epidemic, bear a significant burden and may be more vulnerable to psychological issues compared to the general population. For instance, during the early stages of the epidemic in China, studies revealed that 37% of the general population experienced anxiety [7]. In contrast, healthcare workers showed even higher prevalence rates, with 45% suffering from anxiety [8]. An umbrella review synthesizing the prevalence of anxiety and depression among healthcare workers during the COVID-19 pandemic analyzed ten systematic reviews, incorporating data from 169,157 healthcare professionals across 35 countries. The findings highlighted significant mental health challenges, with anxiety prevalence rates ranging from 17% to 19.8% among physicians and from 22.8% to 27% among nurses [9].

The pandemic affected the mental health of healthcare workers in diverse ways, depending on individual characteristics and specific circumstances [10]. Factors such as gender, profession, age, workplace, and department, along with health and psychological aspects like limited social support and low self-efficacy, have contributed to heightened levels of mental health problems [11]. Furthermore, pre-existing challenges such as underfunded health systems and limited mental health infrastructure have intensified the mental health impact of the pandemic on healthcare workers [12].

To the authors’ knowledge, a significant research gap exists regarding the mental health issues — especially anxiety and stress — faced by Vietnam’s healthcare professionals during the initial wave of the Omicron variant in 2022 [13]. Unlike previous variants, Omicron exhibited increased transmissibility, leading to breakthrough infections even among individuals with multiple vaccine doses [14]. Given the heightened concerns about infection during this wave, it was conceivable that psychological ramifications, including frustration, irritability, burnout, and insomnia, would become widespread due to prolonged social isolation and quarantine measures. In this paper, we investigated the prevalence and factors associated with anxiety and stress among health workers in a Vietnamese hospital during the Omicron wave in 2022 in Vietnam.

## MATERIAL AND METHODS

### Study design and setting

A cross-sectional study was conducted at Hanoi Medical University Hospital, located in the capital city of Hanoi, Vietnam. Established in 2007, the hospital has 700 beds distributed across 44 clinical departments and centers, and it is managed by over 1,100 experienced healthcare professionals. As a training ground for medical education, the hospital serves a diverse range of learners, including undergraduate and graduate students, doctoral candidates, residents, and specialists. Officially endorsed by the Vietnam Ministry of Health, the hospital has the authority to establish a network of satellite medical facilities and provide guidance to district-level hospitals nationwide. In addition to its core medical activities, the hospital actively contributes to community health initiatives, such as annual charitable medical examinations and the provision of essential medications in underserved areas, including those with ethnic minority populations. This commitment has garnered the hospital significant recognition, support, and collaboration from the broader community.

During the COVID-19 pandemic, Hanoi Medical University Hospital provided critical support to provinces and cities nationwide, including human and material resources. Simultaneously, the hospital diligently maintained rigorous epidemic prevention and control measures within its premises. In response to the evolving COVID-19 situation in the northern regions, the hospital established a dedicated subsidiary specializing in COVID-19 treatment.

### Study participants

The study participants were health workers at Hanoi Medical University Hospital, encompassing physicians, pharmacists, and nurses actively engaged in practice during the study period. The exclusion criteria included healthcare workers with self-reported histories of depression, anxiety, or other pre-existing psychological illnesses, as well as those currently undergoing treatment with medication for psychological conditions.

### Sample size

During the data collection period, the workforce at Hanoi Medical University Hospital comprised 1,048 employees. The sample size was calculated based on the highest recent prevalence of mental health recorded among healthcare workers in COVID-19 field hospitals in a previous Vietnamese study [15] using the OpenEpi tool [16].

### Data collection

Data collection was conducted using a structured Vietnamese-language questionnaire distributed via a Google survey. The questionnaire was shared through the clinical departments and centers involved in COVID-19 response at Hanoi Medical University Hospital. As a preliminary step, a pilot study was conducted to evaluate the feasibility of administering the survey on a larger scale. Three field experts independently assessed the validity of the questionnaire, leading to several modifications based on their recommendations. Additionally, ten participants were recruited for the pilot study from February 10 to 15, 2022, to ensure the clarity of the questions and eliminate ambiguities. Following this, responsible individuals facilitated the distribution of the survey link to all health workers via social media platforms on February 28, 2022. After reviewing the information page and consenting to participate, respondents could proceed with the questionnaire. To achieve a strong response rate, the survey was distributed multiple times, and the link remained accessible until April 14, 2022.

### Measurements

The DASS-21 Depression, Anxiety, and Stress Scale-21 (DASS-21) [17] was used in this study. The DASS-21 is a self-report questionnaire comprising 21 items, organized into three subscale domains, each containing seven items focused on measuring depression, anxiety, and stress. Participants assessed the extent to which they had encountered each symptom within the preceding week, utilizing a four-point response scale: 0 = never, 1 = sometimes, 2 = frequently, and 3 = most or all of the time. Subscale scores were determined by summing the individual item scores, with a potential maximum sum of 21 for each subscale. The final score for each subscale was multiplied by 2 to assess the extent of negative emotional status. Heightened scores indicated increased levels of mental health problems, spanning from mild to extremely severe manifestations. In this paper, we only report the anxiety and stress dimensions. Participants were divided into two groups based on their DASS-21 scores: those with symptoms of anxiety (≥8) or stress (≥15) and those without symptoms (scores <8 for anxiety and <15 for stress). The level of anxiety was classified as normal (0-7), mild (8-9), moderate (10-14), severe (15-19), and extremely severe (≥20). The degree of stress was categorized as normal (0-14), mild (15-18), moderate (19-25), severe (26-33), and extremely severe (≥34) [18][19].

In this study, the Vietnamese version of the DASS-21 demonstrated strong internal consistency reliability, with an overall Cronbach’s α = 0.953, and for its subscales: anxiety (Cronbach’s α = 0.858) and stress (Cronbach’s α = 0.897).

For independent variables, we categorized demographic attributes, encompassing age (<30, 30-40, and >40), gender (men and women), marital status (single, married, and divorced), and working years (< 5 years, 5 - 10 years, and > 10 years).

Beyond demographic attributes, the study collected various associated factors, such as the working conditions experienced by health workers during their involvement in anti-epidemic efforts (including workload, remuneration regime, and occupational roles involving direct contact with COVID-19 patients), colleague support, experiences of discriminatory behavior from relatives and friends, and encounters with discourteous patients.

### Statistical analysis

Data were exported from Google Forms to Excel and then imported into SPSS version 22.0 for analysis. To visualize the data, we utilized RStudio software version 4.2.0. First, data cleaning was conducted to identify errors and remove incorrect, incomplete, irrelevant, duplicated, or improperly formatted data. Only data from respondents who completed all survey items were included in the analysis. Among the total 812 respondents, 754 provided fully completed responses. Second, descriptive statistics (including percentages, means, and standard deviations) were employed to analyze demographic characteristics and responses to the survey questions. Chi-square tests were conducted to assess disparities in the prevalence of surveyed symptoms across two or more distinct groups. Third, unadjusted binary logistic regression analysis was used to identify potential factors correlated with the presence of these symptoms among health workers. The corresponding unadjusted odds ratios (ORs) and 95% confidence intervals (95% CIs) were calculated to investigate the relationships between each factor and the respective outcomes. Finally, multinomial logistic regression was employed to compute adjusted ORs and their corresponding 95% CIs. This model was used to adjust for confounding variables such as gender, age, years of professional experience, and marital status. All statistical analyses conducted in this study adhered to a two-tailed approach, considering a *P* value of less than 0.05 as statistically significant.

### Patient and public involvement

Patients or members of the public were not involved in the study design, formulation of research questions, interpretation of results, or reporting of the research.

## RESULTS

### Demographic characteristics

A total of 754 healthcare workers participated in the study, with a mean age of 32.4 ± 6.5 years. Most participants were aged between 23 and 30 years (46.3%), followed by those aged 31 to 40 years (44.2%). In terms of gender, female participants comprised the majority at 73.2% of the total sample. Most respondents (44.4%) reported having a tenure of 1 to 5 years in their current hospital position. Additionally, a significant proportion of health workers (52.3%) were directly involved in patient care or held roles requiring direct contact with individuals diagnosed with COVID-19.

### The prevalence of anxiety and stress among health workers

**Figure 1:**
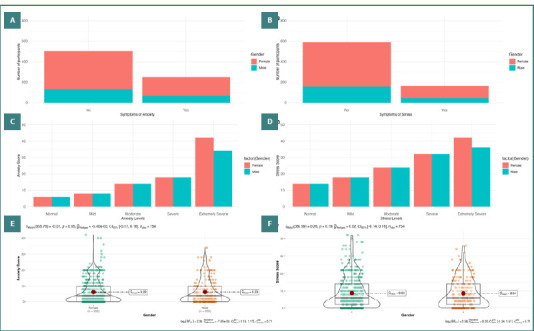
Gender-based distribution of anxiety and stress among Vietnamese health workers using the DASS-21 tool

Figure 1(A-F) and Table S1 depict the prevalence of symptoms related to anxiety and stress among Vietnamese health workers during the initial Omicron waves. Anxiety symptoms were noted in 33% (95% CI, 29.4–36.7) of health workers, with extremely severe levels observed in 10.9%, severe in 4.1%, moderate in 12.9%, and mild in 5.2% of instances (Figure 1A and 1C). The prevalence of stress was 22% (95% CI, 19.2–24.8), with extremely severe manifestations in 2%, severe in 2.8%, moderate in 10.6%, and mild in 6.6% of cases (Figure 1B and 1D). Figures 1E and 1F indicate no significant differences between male and female frontline health workers in the reporting scores for anxiety and stress (*P* = 0.799 and *P* = 0.948, respectively). However, women had higher scores than men at extremely severe levels (Figure 1C and 1D).

Appendix 1

### Univariate and multiple regression analysis of factors associated with mental health problems

Table 1 presents the results of our univariate analysis, which indicated no significant associations between demographic characteristics and symptoms of stress. In contrast, variables that showed statistically significant associations with anxiety in univariate logistic regressions included the age group over 40 (OR univariable = 1.89; 95% CI, 1.06–3.41), individuals with over 10 years of work experience (OR univariable = 1.46; 95% CI, 1.00–2.13), and married health workers (OR univariable = 1.51; 95% CI, 1.08–2.09). However, multivariate regression analysis did not confirm significant associations between demographic characteristics and symptoms of anxiety and stress among health workers (data not shown).

**Table 1:** Univariate analysis of demographic characteristics associated with self-reported anxiety and stress among health workers

General characteristics		Anxiety		Stress	
		*P*	OR 95% CI	*P*	OR 95% CI
Gender	Women	0.689	1	0.917	1
	Men		1.07 (0.76–1.51)		1.021 (0.693–1.505)
Age	< 30		1		1
	30 - 40	0.09	1.31 (0.951–1.80)	0.256	1.232 (0.859–1.767)
	> 40	0.03*	**1.89** **(1.06–3.41)**	0.158	1.610 (0.827–3.134)
Working years	< 5 years		1		1
	> 5 - 10 years	0.482	1.14 (0.79–1.64)	0.257	1.270 (0.840–1.922)
	> 10 years	0.047*	**1.46** **(1.00–2.13)**	0.065	1.497 (0.974–2.301)
Marital status	Single		1		1
	Married	0.015*	**1.51** **(1.08–2.09)**	0.191	1.284 (0.882–1.869)
	Divorced	0.306	1.98 (0.52–754)	0.993	1.008 (0.263–3.855)

Table 2 shows the results obtained from the multivariable logistic regression analysis, revealing a significant association between direct contact with COVID-19 patients and an elevated likelihood of the health workers experiencing symptoms of stress. Specifically, the odds ratio for stress was 2.12 (*P* < 0.01, 95% CI, 1.447–3.092). Health workers who were exposed to a higher prevalence of discriminatory behavior in their interactions with relatives and friends were found to be associated with increased odds of exhibiting symptoms related to anxiety. Specifically, the odds ratio for anxiety was notably higher at 2.453 (*P* < 0.001, 95% CI, 1.755–3.428). Similarly, heightened workload significantly increased the risk of healthcare workers experiencing anxiety (*P* < 0.001, 95% CI, 1.38–2.75). Moreover, the absence of adequate support from colleagues emerged as a significant contributing factor to stress among health workers.

**Table 2:** Multiple regression analysis of factors associated with self-reported anxiety and stress among Vietnamese health workers

Factors		OR	*P*	95% CI
**Anxiety**				
Experiencing discriminatory behavior from relatives and friends	Yes	2.453	<0.001	1.755–3.428
	No	1		
Heightened workload	No	1	<0.001	1.38–2.75
	Yes	1.949		
Appropriateness of the remuneration regime	Yes	1	0.002	1.378–4.039
	No	2.359		
**Stress**				
Encounters with discourteous patients	Yes	1.585	0.046	1.008–2.494
	No	1		
Occupational role involving direct contact with COVID-19 patients	Yes	2.115	<0.001	1.447–3.092
	No	1		
Colleague support	Yes	1	0.036	1.071–7.170
	No	2.771		

## DISCUSSION

Anxiety and stress were common mental health issues studied during the COVID-19 pandemic [20,21]. While their symptoms can overlap, they differ significantly. Stress involves irritability, anger, fatigue, muscle pain, digestive issues, and difficulty sleeping. Anxiety is characterized by persistent, excessive worries and similar symptoms to stress, including insomnia, difficulty concentrating, fatigue, muscle tension, and irritability [22]. These mental health disorders represent a significant component of the global disease burden, with a notably high prevalence in Southeast Asian nations, including Vietnam [23]. The advent of the COVID-19 pandemic has engendered an unparalleled psychological strain upon the medical workforce, particularly for those on the front lines who were in direct contact with infected patients. In this context, frontline healthcare workers at Hanoi Medical University Hospital experienced increased stress from working in temporary facilities designed to accommodate the surge of patients from intensive care units. These conditions were often compounded by inadequate protective equipment. Additionally, they frequently covered extra shifts to make up for colleagues absent due to sickness or quarantine and rapidly adapted to new medical procedures. They also faced complex clinical and ethical decisions that critically impacted patient outcomes, often under heightened mortality rates. Therefore, this investigation aimed to elucidate the prevailing burden of symptoms pertaining to anxiety and stress experienced by healthcare practitioners in Vietnam during the inaugural phase of the Omicron wave in the late spring of 2022.

Previous research has indicated that mental health outcomes following COVID-19 infection exhibit a dynamic trajectory over time. In Vietnam, which has experienced four waves of the COVID-19 pandemic, the prevalence of mental health issues among medical staff has varied according to the progression of each wave. During the second wave, a study conducted by Anh Le Thi Ngoc between July and August 2020 involving healthcare workers at COVID-19-designated hospitals in southern Vietnam identified that 11.5% showed signs of anxiety, and 7.7% demonstrated symptoms of stress, with most cases being mild to moderate in severity [18]. In contrast, during the third wave, Ha Thi Thu Tran's investigation of healthcare workers in Ho Chi Minh City, conducted from July 15 to September 25, 2021, reported significantly higher prevalence rates of anxiety (38.3%) and stress (60.2%) [24]. However, the fourth wave, which began on April 27, 2021, primarily driven by the Delta variant (formerly known as the Indian variant), dramatically altered the situation. This wave represented the most severe and deadly stage, with the highest mortality rate. In a study by Bach Tran from October to November 2021, which utilized a more representative sample, results indicated that 34.0% reported moderate anxiety symptoms, and 49.3% reported elevated stress levels, reflecting a further exacerbation of mental health problems compared to earlier waves [15]. Since November 2021, the novel SARS-CoV-2 Omicron variant has replaced the Delta variant as the most prevalent strain globally, characterized by lower severity and mortality, particularly among vaccinated individuals [25]. This shift may have also influenced the incidence of mental health problems among healthcare workers. In our study cohort, we observed notably reduced rates of anxiety and stress compared to previous COVID-19 waves in Vietnam. Specifically, 33.1% showed symptoms of anxiety, and 21.6% reported stress during the initial wave of the Omicron subvariant in northern Vietnam. This pattern aligns with research by Hien Thu Pham, which found that among Vietnamese hospital staff in July 2022, the prevalence of symptoms was 24.7% for anxiety and 13.9% for stress [19].

Although this observation is consistent with a logical interpretation of an ongoing pandemic, it has not yet been investigated or definitively proven, nor was such a minimal impact anticipated in Vietnam. Hence, a plausible hypothesis emerges suggesting that the impact of the novel Omicron subvariant on the psychological well-being of Vietnamese health workers might exhibit a diminished magnitude compared to its effect in the earlier phases of the pandemic. Several factors may elucidate this observed phenomenon. Firstly, the mortality rate associated with the Omicron variant was significantly lower, as evidenced in Vietnam, where April 2022 data showed a weekly fatality rate of 0.002%, in contrast to a global average of 0.004% [25]. Secondly, improvements in vaccination programs, COVID-19 screening procedures, treatment methods, and increased public awareness have collectively reduced the virulence of the virus and lessened public fear concerning COVID-19. Lastly, healthcare professionals have adapted to the ongoing COVID-19 situation by interacting with diverse affected social cohorts. Their extensive professional experience enhances their comprehension and assessment of disease-related information and ensures access to adequate personal protective equipment and specialized infection control expertise. Compared to the general population [23], our study showed that the mental health of frontline healthcare workers has been significantly and adversely affected since the pandemic began. This highlights the need for greater recognition and more effective support for their specific needs. However, despite improvements in medical infrastructure and widespread vaccine distribution, healthcare workers continue to face persistent psychological challenges. This situation may be partly attributed to their continuous frontline involvement, which could make them overlook changes in their physical and psychological states amidst ongoing work-related stress. This could have also led to a psychological coping mechanism, while the fatigue stemming from detrimental psychological impacts and the subsequent emergence of mental symptoms may have manifested subsequently [20].

Our multivariate analysis aimed at identifying predictors of anxiety and stress offers vital insights for targeted interventions to address mental health challenges among Vietnamese healthcare workers during the COVID-19 pandemic. We confirmed the significance of factors like direct contact with COVID-19 patients, supportive interactions with colleagues, and discriminatory behavior from relatives and friends in influencing these conditions. These findings align with precedent studies that have established associations between mental health problems in medical personnel and factors such as direct exposure to COVID-19 patients [21], experiences of discrimination [26,27], and the presence of supportive interactions from colleagues [28]. These findings indicate that a significant proportion of the substantial cohort seeking psychological assistance comprises health workers. It is crucial, therefore, to prioritize their mental well-being by providing proactive psychological counseling and support, which could improve mental health standards within this group. Additionally, society should ensure widespread psychological care for the general public through robust health education initiatives that increase disease awareness and reduce public anxiety. Our results suggest that special attention should be directed towards all frontline health workers, necessitating comprehensive engagement from mental health professionals, hospital administrators, and society at large. Lastly, our study did not reveal any significant correlations between various demographic characteristics and anxiety and stress levels, contrasting with prior studies that identified age and gender as factors [29,30]. This discrepancy highlights the need for further thorough research in this critical area.

We acknowledge several potential limitations in our study. Firstly, our study has published depression-related results in the paper titled “Symptoms of depression among healthcare workforce and some factors related in covid-19 at Hanoi Medical University Hospital in 2022” [31]. Secondly, the use of the DASS-21 as a self-assessment measure for anxiety and stress introduces inherent biases and potential errors. Despite its widespread application in clinical settings, it is important to note that the DASS-21 is not a diagnostic tool. Consequently, in our study, we did not perform any objective clinical assessments to definitively confirm the presence of these conditions among healthcare workers. Additionally, the cross-sectional design of our study prevents us from establishing causality. Conducting the study in a single center limits the generalizability of our findings to other settings. The assessment of psychological statuses, including anxiety and stress, relied on subjective measures, which could introduce inaccuracies. Thirdly, we relied on previously established cutoff points used for Vietnamese healthcare workers during the COVID-19 pandemic. The absence of specific cutoff points in the DASS-21 for our study population may lead to the risk of misinterpretation of psychological outcomes. This can result in the possible overestimation or underestimation of an individual's psychological status when using these cutoffs to define psychological categories. Therefore, it is important to interpret the present findings with caution. Finally, the voluntary nature of participant recruitment in our study may introduce response bias, given that individuals who opted to participate could significantly differ from those who declined.

### Implications

While the pandemic may have subsided, further research is needed to identify any long-term psychological effects that may persist among medical professionals. Therefore, ongoing studies should track the evolution of mental health issues during and after the pandemic, and effective measures should be implemented to prevent and alleviate mental health deterioration in healthcare workers.

Despite numerous recommendations on combating burnout and adverse mental health outcomes among healthcare workers, there was limited evidence of their effectiveness during the pandemic [32]. Our findings underscore the urgent need for interventions and policies to reduce the prevalence of mental health problems among healthcare workers, even as the pandemic subsides. One approach could involve establishing well-resourced internal mental well-being departments at the hospital level, with designated welfare champions across various departments and healthcare roles. Hospitals should ensure that counseling and peer support services are widely accessible and that barriers to access are minimal. Timely implementation of appropriate psychological interventions is crucial during crises to support healthcare personnel, preserving their mental well-being and work efficacy under demanding conditions.

Our study found that increased perception of COVID-19-associated discrimination was linked to poorer mental health among healthcare workers. Therefore, further research should identify resilience as a protective factor against the negative impacts of public discrimination toward frontline healthcare workers. Organizational strategies to mitigate the effects of discrimination during the pandemic should be prioritized.

Moreover, our study also found that healthcare workers faced high workloads and increased anxiety during the Omicron COVID-19 pandemic. To address these challenges, healthcare policymakers and hospital management should set clear mental health targets, standardize measurement tools to assess and monitor workloads, and increase human resource capacity to ensure that staff are not excessively strained. Optimizing work processes and adopting digital technologies can also help reduce administrative burdens [33]. Promoting stable teams, providing clear guidelines, and ensuring the availability of social support are also crucial. Finally, implementing flexible working arrangements can facilitate better work-life balance and improve the overall health and well-being of healthcare workers.

## CONCLUSION

In summary, it was evident that Vietnamese health workers continued to grapple with mental health issues during the Omicron wave, albeit at a decreased prevalence compared to previous COVID-19 outbreaks. The findings underscore a robust association between the mental well-being of Vietnamese health workers and a diverse array of factors, encompassing both the working environment and determinants related to discrimination. Despite longstanding concerns regarding mental health issues among healthcare workers, the COVID-19 pandemic has intensified these challenges, even amidst heightened awareness and support measures. As we transition to a state of COVID endemicity, there is a potential for a subsequent mental health crisis among this population. Therefore, we urge healthcare systems to conduct comprehensive audits of their workers' mental health status and to continually evaluate contributing factors. This approach will guide the implementation of formal measures to protect healthcare workers and ensure the sustainability of healthcare delivery.

## Data Availability

The datasets generated during and/or analyzed during the current study are available from the corresponding author upon reasonable request.

## References

[1] World Health Organization (2023). Coronavirus disease (COVID-19) pandemic. World Health Organization [Internet].

[2] Kessler RC, Ruhm CJ, Puac-Polanco V, Hwang IH, Lee S, Petukhova M V (2022). Estimated Prevalence of and Factors Associated With Clinically Significant Anxiety and Depression Among US Adults During the First Year of the COVID-19 Pandemic. JAMA Netw Open.

[3] Cai H, Tu B, Ma J, Chen L, Fu L, Jiang Y (2020). Psychological Impact and Coping Strategies of Frontline Medical Staff in Hunan Between January and March 2020 During the Outbreak of Coronavirus Disease 2019 (COVID 19) in Hubei, China. Med Sci Monit.

[4] Rosenberg AR (2020). Cultivating Deliberate Resilience During the Coronavirus Disease 2019 Pandemic. JAMA Pediatr.

[5] Salari N, Hosseinian-Far A, Jalali R, Vaisi-Raygani A, Rasoulpoor S, Mohammadi M (2020). Prevalence of stress, anxiety, depression among the general population during the COVID-19 pandemic: a systematic review and meta-analysis. Global Health.

[6] James SL, Abate D, Abate KH, Abay SM, Abbafati C, Abbasi N (2018). Global, regional, and national incidence, prevalence, and years lived with disability for 354 diseases and injuries for 195 countries and territories, 1990-2017; a systematic analysis for the Global Burden of Disease Study 2017.. The Lancet.

[7] Wang C, Pan R, Wan X, Tan Y, Xu L, Ho CS (2020). Immediate Psychological Responses and Associated Factors during the Initial Stage of the 2019 Coronavirus Disease (COVID-19) Epidemic among the General Population in China. International Journal of Environmental Research and Public Health.

[8] Lai J, Ma S, Wang Y, Cai Z, Hu J, Wei N (2020). Factors Associated With Mental Health Outcomes Among Health Care Workers Exposed to Coronavirus Disease 2019. JAMA Netw Open.

[9] Fernandez R, Sikhosana N, Green H, Halcomb EJ, Middleton R, Alananzeh I (2021). Anxiety and depression among healthcare workers during the COVID-19 pandemic: a systematic umbrella review of the global evidence. BMJ Open.

[10] Saragih ID, Tonapa SI, Saragih IS, Advani S, Batubara SO, Suarilah I (2021). Global prevalence of mental health problems among healthcare workers during the Covid-19 pandemic: A systematic review and meta-analysis. Int J Nurs Stud.

[11] Chew NWS, Lee GKH, Tan BYQ, Jing M, Goh Y, Ngiam NJH (2020). A multinational, multicentre study on the psychological outcomes and associated physical symptoms amongst healthcare workers during COVID-19 outbreak. Brain Behav Immun.

[12] Maliwichi L, Kondowe F, Mmanga C, Mchenga M, Kainja J, Nyamali S (2024). The mental health toll among healthcare workers during the COVID-19 Pandemic in Malawi. Sci Rep.

[13] Thanh PT, Bao NT, Thi PQ-G, Thanh TV, Trong HT, Phuoc PS (2022). Incidence of SARS-CoV-2 Infection during the Omicron Variant Emergence in Southern Vietnam: Prior Infection versus Third-Dose Vaccination. Microbiol Spectr.

[14] Al-Tawfiq JA, Hoang V-T, Le Bui N, Chu D-T, Memish ZA (2022). The Emergence of the Omicron (B.1.1.529) SARS-CoV-2 Variant: What is the Impact on the Continued Pandemic?. Journal of epidemiology and global health. Switzerland.

[15] Tran B, Nguyen MT, Auquier P, Boyer L, Fond G, Vu GT (2023). Psychological impacts of COVID-19 on Vietnamese health workers over the prolonged restricted COVID-19 responses: a cross-sectional study. BMJ Open.

[16] Dean AG, Sullivan KM SM (2023). OpenEpi: Open Source Epidemiologic Statistics for Public Health. Version. http://www.OpenEpi.com.

[17] Lovibond SH (1995). Manual for the depression anxiety stress scales. Sydney psychology foundation.

[18] Le Thi Ngoc A, Dang Van C, Nguyen Thanh P, Lewycka S, Van Nuil JI (2022). Depression, anxiety, and stress among frontline health workers during the second wave of COVID-19 in southern Vietnam: A cross-sectional survey. PLOS global public health.

[19] Thu Pham H, Viet Cao T, Bich Le N, T-T Nguyen N, Thi Ngoc Vuong B, Vu Dieu Pham L (2023). Depression, anxiety and stress among healthcare workers in the context of the COVID-19 pandemic: a cross-sectional study in a tertiary hospital in Northern Vietnam. Front Public Health.

[20] Chen B, Li Q-X, Zhang H, Zhu J-Y, Yang X, Wu Y-H (2022). The psychological impact of COVID-19 outbreak on medical staff and the general public. Curr Psychol.

[21] Tong J, Zhang J, Zhu N, Pei Y, Liu W, Yu W (2022). Effects of COVID-19 pandemic on mental health among frontline healthcare workers: A systematic review and meta-analysis. Frontiers in psychology.

[22] American Psychological Association (2019). What’s the difference between stress and anxiety?. https://www.apa.org/topics/stress/anxiety-difference.

[23] Tran QD, Vu TQC, Phan NQ (2022). Depression prevalence in Vietnam during the Covid-19 pandemic: A systematic review and meta-analysis. Ethics Med Public Health.

[24] Tran HTT, Nguyen YH, Vuong TD, Bui L Van, Doan HT, Le HTT (2023). High Prevalence of Post-Traumatic Stress Disorder and Psychological Distress Among Healthcare Workers in COVID-19 Field Hospitals: A Cross-Sectional Study from Vietnam. Psychol Res Behav Manag.

[25] Doan LP, Le Vu MN, Vu GT, Le HT, Nguyen LH, Latkin CA (2023). The COVID-19 endemic in Vietnam: Contextual considerations and implications. Frontiers in Public Health.

[26] Badrfam R, Qorbani M, Zandifar A (2022). Status of stigma on the health care workers related to COVID-19 at the first wave of the pandemic in Iran: A qualitative study. Front Psychiatry.

[27] Nabavian M, Rahmani N, Seyed Nematollah Roshan F, Firouzbakht M (2023). Nurses’ experiences of the social stigma caused by the COVID-19 pandemic: a qualitative study. J Res Nurs.

[28] Styra R, Hawryluck L, McGeer A, Dimas M, Lam E, Giacobbe P (2022). Support for health care workers and psychological distress: thinking about now and beyond the COVID-19 pandemic. Health Promot Chronic Dis Prev Can.

[29] Jassim G, Jameel M, Brennan E, Yusuf M, Hasan N, Alwatani Y (2021). Psychological Impact of COVID-19, Isolation, and Quarantine: A Cross-Sectional Study. Neuropsychiatr Dis Treat.

[30] Gabarrell-Pascuet A, Koyanagi A, Felez-Nobrega M, Cristóbal-Narváez P, Mortier P, Vilagut G (2023). The Association of Age With Depression, Anxiety, and Posttraumatic Stress Symptoms During the COVID-19 Pandemic in Spain: The Role of Loneliness and Prepandemic Mental Disorder. Psychosom Med.

[31] Sharifi M, Asadi-Pooya AA, Mousavi-Roknabadi RS (2021). Burnout among Healthcare Providers of COVID-19; a Systematic Review of Epidemiology and Recommendations. Arch Acad Emerg Med.

[32] Sidi H (2020). The Psychological Sequelae during Mental Health and COVID-19 Pandemic: Learning from the Past for Today’s Coping Styles. Med Health.

