# Supplementary material for: Anxiety and stress among Vietnamese health workers during the emergence of the SARS-CoV-2 Omicron variant: a cross-sectional study
Source: J Med Life. 2024 Oct;17(10):940–7. doi: 10.25122/jml-2024-0275 (PMC11665749; doi:10.25122/jml-2024-0275)
Supplement: Supplementary file 1 [file JMedLife-17-940-s001.pdf]

Appendix 1

Table S1. Gender-based distribution of anxiety and stress among Vietnamese health workers using the DASS-21 tool

	n	%	Female	Male	*P*	Scores (Mean [SD])
n			552	202		
Age (mean [SD])			32.05 (6.03)	33.50 (7.60)	0.006	
Stress score (mean [SD])			8.83 (9.08)	8.64 (8.36)	0.799	
Indicators of stress symptoms						
No	588	78.00%	431 (78.1%)	157 (77.7%)		
Yes	166	22.00%	121 (21.9%)	45 (22.3%)		
Stress levels (%)						
Normal	588	78.00%	431 (78.1%)	157 (77.7%)		4.84 (4.54)
Mild	50	6.60%	31 (5.6%)	19 (9.4%)		17.1 (1.00)
Moderate	80	10.60%	62 (11.2%)	18 (8.9%)		22.1 (1.15)
Severe	21	2.80%	15 (2.7%)	6 (3.0%)		28.4 (1.75)
Extremely Severe	15	2.00%	13 (2.4%)	2 (1.0%)		37.3 (2.99)
Overall	754					8.78 (8.89)
Anxiety score (mean (SD))			6.29 (7.47)	6.33 (7.51)	0.948	
Indicators of anxiety symptoms						
No	505	67.00%	372 (67.4%)	133 (65.8%)		
Yes	249	33.00%	180 (32.6%)	69 (34.2%)		
Anxiety levels (%)						
Normal	505	67.00%	372 (67.4%)	133 (65.8%)		1.85 (1.97)
Mild	39	5.20%	25 (4.5%)	14 (6.9%)		8.00 (0)
Moderate	97	12.90%	72 (13.0) %	25 (12.4%)		11.7 (1.62)
Severe	31	4.10%	23 (4.2%)	8 (4.0%)		16.9 (1.01)
Extremely Severe	82	10.90%	60 (10.9%)	22 (10.9%)		22.5 (5.00)
Overall	754					6.30 (7.48)

© 2024 by the authors. This article is an open access article distributed under the terms and conditions of the Creative Commons Attribution (CC BY 4.0) license.
